# Papilledema is not the point: intracranial pressure dysregulation as a common pathway in migraine and idiopathic intracranial hypertension

**DOI:** 10.1093/braincomms/fcag259

**Published:** 2026-07-07

**Authors:** Simone Braca, Giuseppe Cardillo, Lorenzo Ugga, Serena Capasso, Antonio Meo, Angelo Miele, Mattia Sansone, Guido Ferra, Angelo Ranieri, Cinzia Valeria Russo, Antonio Stornaiuolo, Gennaro Cretella, Caterina Giannini, Roberto De Simone

**Affiliations:** Headache Centre, Department of Neurological Sciences, Reproductive Sciences and Odontostomatology, University of Naples ‘Federico II’, 80131 Naples, Italy; MEDyLAB s.r.l., Bologna 40124, Italy; Department of Advanced Medical and Surgical Sciences, University of Campania ‘Luigi Vanvitelli’, 80138 Naples, Italy; Department of Advanced Medical and Surgical Sciences, University of Campania ‘Luigi Vanvitelli’, 80138 Naples, Italy; Neurology Unit, Department of Translational Medicine, University of Eastern Piedmont Amedeo Avogadro School of Medicine, 28100 Novara, Italy; Headache Centre, Department of Neurological Sciences, Reproductive Sciences and Odontostomatology, University of Naples ‘Federico II’, 80131 Naples, Italy; Department of Neurology and Stroke Unit, Headache Centre, ‘San Giuseppe Moscati’ Hospital, Aversa 81031, Italy; Headache Centre, Department of Neurological Sciences, Reproductive Sciences and Odontostomatology, University of Naples ‘Federico II’, 80131 Naples, Italy; Division of Neurology and Stroke Unit, ‘Antonio Cardarelli’ Hospital, 80131 Naples, Italy; Headache Centre, Department of Neurological Sciences, Reproductive Sciences and Odontostomatology, University of Naples ‘Federico II’, 80131 Naples, Italy; Headache Centre, Department of Neurological Sciences, Reproductive Sciences and Odontostomatology, University of Naples ‘Federico II’, 80131 Naples, Italy; Headache Centre, Department of Neurological Sciences, Reproductive Sciences and Odontostomatology, University of Naples ‘Federico II’, 80131 Naples, Italy; Headache Centre, Department of Neurological Sciences, Reproductive Sciences and Odontostomatology, University of Naples ‘Federico II’, 80131 Naples, Italy; Headache Centre, Department of Neurological Sciences, Reproductive Sciences and Odontostomatology, University of Naples ‘Federico II’, 80131 Naples, Italy

**Keywords:** idiopathic intracranial hypertension, idiopathic intracranial hypertension without papilledema, migraine physiopathology, sinus stenosis, papilledema

## Abstract

Accumulating evidence challenges the traditional distinction between migraine and idiopathic intracranial hypertension (IIH). We tested whether impaired CSF volume/pressure regulation driven by dural venous sinus stenosis represents a shared substrate. Brain MRI and magnetic resonance venography scans from 161 consecutive patients—migraine without aura (MwoA, *n* = 89), migraine with aura (MwA, *n* = 36) and IIH (*n* = 36)—were retrospectively analysed and compared with 37 prospectively recruited healthy controls. An Extended Combined Conduit Score—designed to quantify the degree and extent of venous-sinus narrowing, with higher values indicating greater outflow impairment, and also evaluating the superior sagittal sinus—quantified stenosis. Additional neuroradiological markers of raised intracranial pressure (empty sella, posterior globe flattening and optic nerve sheath diameter) were recorded. A receiver operating characteristic analysis defined the optimal stenosis degree threshold for distinguishing patients from controls, after which prevalence comparisons were performed. Median Extended Combined Conduit Score increased step wise from controls (2, interquartile range 1–3) to MwoA (3, 2–4), MwA (3, 3–4) and IIH (6, 5–6) (*P* < 0.001). Both migraine groups scored higher than controls (*P* < 0.001) and lower than IIH (*P* < 0.001). The area under the receiver operating characteristic curve was 0.786 (95% confidence interval 0.716–0.856); a cut-off ≥3 provided 74.5% sensitivity and 73.0% specificity. Using this threshold, venous stenosis was more frequent in MwoA and MwA than in controls (each *P* < 0.001) and highest in IIH (*P* < 0.001 versus all); MwA exceeded MwoA (*P* = 0.007) and was comparable to IIH (*P* = 0.187). Bilateral stenosis occurred in 0% of controls, nearly 25% of patients with migraine (*P* < 0.001 versus controls) and 77.8% of IIH patients (*P* < 0.001). Empty sella was more common in MwoA (*P* = 0.004) and MwA (*P* = 0.025) than in controls, and markedly enriched in IIH (*P* < 0.001 versus all). Other radiological markers were similarly more prevalent in IIH (all *P* < 0.001) showing no additional significant between-group differences. Findings support a pathogenetic continuum linking MwoA, MwA and IIH, challenging the diagnostic primacy of papilledema, the rarity of IIH without it, and the perceived irrelevance of unilateral sinus narrowing. As already observed in IIH, an impaired intracranial volume/pressure regulation appears a necessary though not sufficient condition for migraine development; a ‘primary’ predisposition to migraine, likely multifactorial and widely variable among individuals and in the same individual over time, is required for typical migraine pain to develop, also modulating its frequency. Future studies should assess whether targeting intracranial pressure, thus potentially preventing calcitonin gene-related peptide release, may complement or substitute calcitonin gene-related peptide based therapies in migraine patients.

## Teaser

De Simone et al. compared MRI scans from migraine and idiopathic intracranial hypertension patients versus controls. Venous sinus stenosis and other pressure-related markers, hallmarks of intracranial hypertension, were also common in migraine, suggesting shared intracranial pressure dysregulation. Papilledema appeared only in the severest stenosis cases, likely representing the disease’s end-spectrum.

## Graphical Abstract

**Graphical Abstract:**
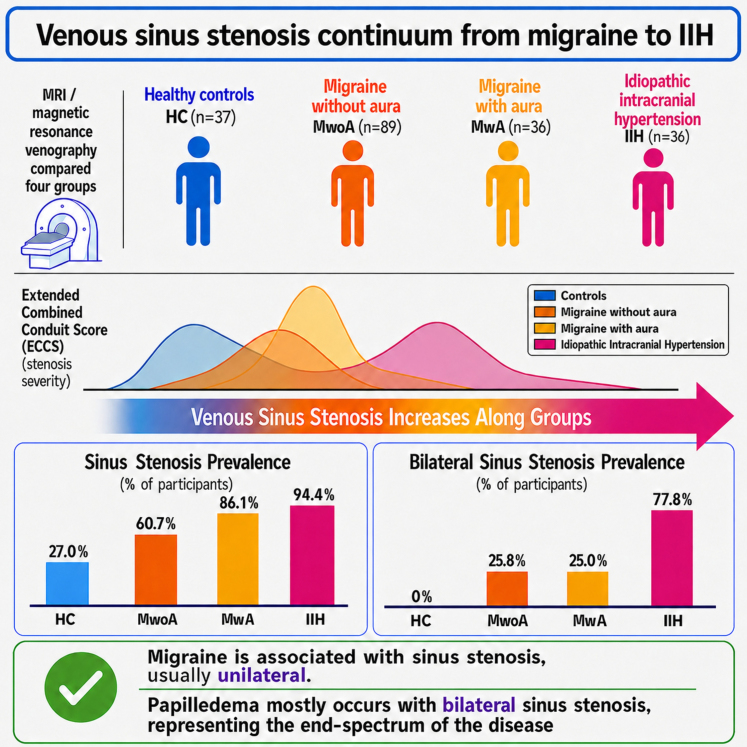

## Background

Migraine is the most common primary headache disorder. According to a recent systematic review of US studies it has a prevalence of 11.7% to 14.7% in the general population, significantly more frequent in females (17.1% to 19.2%) than in males (5.6% to 7.2%).^1^ Although its causes remain unknown, ongoing research points to dysfunctions in central pain processing mechanisms and structures. These dysfunctions are thought to trigger neurogenic inflammation in the peripheral trigemino-vascular system, leading to the abnormal release of pro-inflammatory peptides such as calcitonin gene-related peptide (CGRP) and pituitary adenylate cyclase-activating peptide. These peptides sensitize pain pathways and contribute to migraine attack initiation.^2^ The classification of migraine as a ‘primary’ disorder largely stems from the consistent absence of structural intracranial abnormalities that could be deemed causative.

Idiopathic intracranial hypertension (IIH) is a condition characterized by signs and symptoms of elevated CSF pressure that mimic those seen in space-occupying lesions or cerebral oedema, yet with no identifiable cause.^3^ IIH is a rare disease, affecting 4.7 per 100 000 individuals in the general population with a peak incidence of 15.2 per 100 000 in females of childbearing age.^4^ The mechanisms underlying IIH are not fully understood; however, almost all IIH patients present significant sinus stenosis, a factor increasing the venous pressure upstream. A raised dural sinus pressure due to various aetiologies increases resistance to cerebrospinal fluid reabsorption and is widely accepted as the universal mechanism leading to increased intracranial pressure (ICP).^5^

Papilledema is considered the hallmark of IIH diagnosis.^6^ A variant without papilledema (IIHWOP) has been described.^7^ Although IIHWOP prevalence has never been directly investigated, it is estimated to be less than 6% of IIH cases with papilledema^8^—making it up to twenty times less frequent than the already rare classic IIH. Headache is the most common presenting symptom of IIH, reported in 84% of cases,^9^ and it displays a migraine-like phenotype in nearly 70% of patients. Shared features include severe pulsatile pain aggravated by movement, accompanied by photophobia, phonophobia, nausea and vomiting rendering it clinically indistinguishable from migraine.^7,8^

The similarities between migraine and IIH extend beyond clinical presentation. The two conditions share risk factors such as female sex and sleep disturbances, obesity being a major risk factor for IIH and a known contributor to chronification in migraine;^10,11^ a higher prevalence of allodynic symptoms;^12,13^ therapeutic responses to carbonic anhydrase inhibitors such as topiramate;^14^ high prevalence of dural venous sinus stenosis,^15–18^ considered the most reliable neuroradiological marker of IIH;^19^ elevated CSF opening pressure and a prompt and sometimes sustained clinical response to lumbar puncture with CSF withdrawal.^20,21^

Dural sinuses are the most pain-sensitive intracranial structures,^22^ richly innervated by a trigemino-vascular peptidergic network dominated by CGRP.^23^ We hypothesized that the migraine-like headache in IIH results from the stretching of dural sinus wall due to the parallel raise of dural sinus and CSF pressures in collapsible or anatomically restricted dural sinus carriers, and is therefore CGRP-dependent.^24,25^ This hypothesis was soon supported by a study demonstrating the efficacy of erenumab, an anti-CGRP receptor monoclonal antibody effective in migraine, also in IIH-related migraine-like headache.^26^ Elevated circulating CGRP levels were later documented in IIH.^27,28^

More recently, a further intriguing similarity emerged: GLP-1 receptor agonists such as exenatide and liraglutide proved effective in treating both migraine-like IIH headache and high-frequency or chronic migraine, respectively^29,30^ independently of their weight-loss effects. Notably, the GLP-1 receptor is expressed, as shown by previous literature, on the choroid plexus,^31^ and its activation reduces CSF production rates more effectively than acetazolamide or topiramate,^32^ crucially inhibiting CGRP release in chronic migraine models.^33^ There is also evidence that its activation suppresses central sensitization at the level of the trigeminal caudate nucleus.^34^ These findings and considerations further support the possible association between deranged ICP control and migraine.

Despite these striking clinical, pathophysiological and therapeutic overlaps, migraine and IIH continue to be classified as distinct entities. The hypothesis that they might represent variants of a shared condition has not been adequately explored. This is partly due to two widely accepted and mutually reinforcing assumptions in medical literature: papilledema, which is absent in migraine, is regarded as the most reliable indicator of elevated ICP; and, second, IIHWOP is believed to be much less common than the already infrequent IIH with papilledema, making it an implausible contributor to the pathogenesis of widespread migraine.

However, two simple observations challenge these assumptions. First, papilledema is a visible manifestation in an external organ distant from the subarachnoid space, suggesting that it develops only when ICP surpasses a certain threshold, which can vary significantly among individuals and in the same individual over times.^35^ Papilledema may present asymmetrically or unilaterally in about 10% of cases, potentially due to anatomical differences such as optic canal size.^36-38^ Thus, ICP is not the sole determinant of papilledema; other local anatomical factors also contribute, calling into question its central role in diagnosis. Second, it is counterintuitive—and mathematically implausible—that a less specific condition like IIHWOP meeting all diagnostic criteria but one, would be twenty times less common than classic IIH, fully matching the diagnostic criteria set.

These insights imply that papilledema may better reflect the severity rather than the mere presence of intracranial hypertension. Furthermore, the perceived rarity of IIHWOP may be an artefact of underdiagnosis perpetuated by current diagnostic criteria crafted primarily by ophthalmologists rather than neurologists. These criteria were arguably designed to curb the ‘overuse and misuse of the IIHWOP diagnosis’.^6^ Notably, an increasing number of studies^39-42^ highlight the unacceptably low sensitivity of current IIHWOP diagnostic criteria and provide clear evidence of the urgent need for their revision.^43,44^

Therefore, this is a hypothesis driven study aiming to assess whether papilledema is a marker of severity rather than the presence of IIH, and that unrecognized IIHWOP might be pathogenetically involved in migraine. In order to do so, we assessed the prevalence of four validated neuroradiological signs of intracranial hypertension (venous sinus stenosis, empty sella, optic nerve sheath enlargement and globe flattening) and the degree and bilaterality of sinus stenosis across three patient groups (migraine without aura, migraine with aura and IIH) and a healthy control group.

## Methods

### Study design and participants

We performed a single-centre retrospective study to determine whether signs of increased ICP differed between patients with IIH, migraine and healthy controls. All consecutive patients seen between 1 January 2018 and 31 May 2023 with a diagnosis of migraine without aura (MwoA), migraine with aura (MwA) or IIH, defined as per ICHD-3 and modified Dandy criteria,^45,46^ were screened as a service evaluation. Healthy controls were drawn from an independent prospective project on dural-sinus anatomy in healthy subjects; the study was approved by our local ethics committee (protocol 1496/17). Each participant received an alphanumeric pseudonym; only the treating physician held the key to re-identify individuals, ensuring compliance with applicable privacy regulations. Written consent was obtained from all participants according to the Declaration of Helsinki prior to any study procedures.

### Inclusion and exclusion criteria

Participants were included if they met all of the following: availability of an unenhanced MRI, including venography (MRV) acquired on a scanner with field strength ≥1.5 T; venous phase imaged with a three-dimensional phase-contrast (PC-3D) angiographic sequence; fulfilment of ICHD-3 criteria for MwoA, MwA or IIH. Exclusion criteria were: imaging not fulfilling the imaging requirements, or evidence of concomitant intracranial venous pathology (e.g. cerebral venous sinus thrombosis).

Healthy controls were excluded if they had a body mass index greater than 30, age <18 or >60, any history of headaches—even occasional—unless these were episodic and clearly secondary to minor head trauma, dental pain, sinusitis or cervicogenic headache with pain-related restriction of neck mobility. Additional exclusion criteria encompassed a past or present history of vertigo, imbalance, tinnitus, or aural fullness, except when attributed to otitis, occasional alcohol intoxication, or an ear-wax plug; any chronic inflammatory, autoimmune or vascular disease, or other disorders causing chronic pain; arterial hypertension; previous episodes of reduced or lost consciousness; cognitive impairment; glaucoma, diplopia, amaurosis or papilledema; current pregnancy; thrombophlebitis or a documented genetic or acquired thrombophilia; unexplained recurrent epistaxis at any time of life; and claustrophobia that would preclude undergoing MRI examination.

### Clinical data collection

The monthly number of headache days over the previous three months was recorded at the first visit, along with any ongoing preventive treatment, if applicable. As part of our routine work-up, we request an unenhanced MRI/MRV for patients who have never undergone one, to be performed inter-ictally.

### MRI/MRV acquisition and image analysis

Brain and orbit MRI was acquired together with the MRV on 1.5 T or higher scanners. Imaging sequences including sagittal 3-D T1-weighted, axial and coronal fast spin echo 3-D T2-weighted, axial FLAIR and a fat-suppressed high-resolution axial T2 through the orbits were selected. All MRVs were obtained using PC-3D venography. Although sequence parameters (TR/TE, velocity-encoding and voxel size) varied slightly across scanners, they conformed to recognized clinical standards for dural-sinus imaging.^47^

### Extended Combined Conduit Score

The Combined Conduit Score proposed by Farb *et al*. in 2003^48^ quantifies the degree and extent of venous-sinus narrowing, with higher values indicating greater outflow impairment. Since an isolated stenosis of the superior sagittal sinus (SSS) may cause an intracranial hypertension syndrome responsive to stenting,^49^ venous sinus calibre variations were graded using an *Extended Combined Conduit Score* (ECCS) modified by Farb^48^ that included evaluation of the SSS.

Each of the three main sinuses (superior sagittal sinus, right transverse sinus and left transverse sinus) was scored by comparing its minimum diameter to the maximum diameter among the three sinuses near the torcular Herophili. An additional point was added to the sum of dural sinuses score in cases missing torcular confluence, where the superior sagittal sinus drained into one transverse sinus and the straight sinus into the other, preventing pressure compensation of the superficial and deep venous drainage systems at the torcular. Thus, the final score ranged from 0 to 10. The score is summarized in Table 1.

**Table 1:** Extended Combined Conduit Score

Dural sinus calibre	Score^a^
Luminal narrowing <33%	0
Luminal narrowing 33–66%	1
Luminal narrowing >66%	2
Apparent ‘flow gap’	3
Torcular non-confluence^b^	+1

^a^Scores for each sinus were summed to yield a global outflow-compromise score.

^b^One additional point was added when the confluence of sinuses showed torcular non-confluence.

Two expert neuroradiologists, both blinded, independently scored MRI signs and dural sinus calibre according to the scale; in case of disagreement the lower score was recorded to avoid overestimation. Signs of increased ICP, including empty sella, flattening of the posterior ocular globe and optic nerve sheaths diameters, were similarly assessed. Empty sella was investigated on sagittal T1 images and was defined as pituitary gland to sella turcica height ratio <0.5;^50^ posterior globe flattening was judged qualitatively on the orbital T2 sequence and recorded as present whenever the normal posterior convexity of the ocular bulb was lost. Optic-nerve-sheath diameter was measured with electronic callipers perpendicular to the nerve axis exactly 3 mm behind the globe on the same high-resolution T2 images.

### Derivation of a dichotomous venous-stenosis cut-off

To binarize the ECSS, an optimal cut-off distinguishing cases (Groups B-C-D) from healthy controls (Group A) by constructing a receiver operating characteristic curve is to be identified; the statistical procedures are detailed in the appropriate section. Scores meeting or exceeding this threshold will denote ‘venous stenosis present’.

### Bilateral versus unilateral sinus stenosis

Stenoses were classified as *bilateral* when the ECCS reached at least the cut-off and the absolute difference between transverse sinuses was ≤ 1. If the score met the cut-off but the transverse sinus difference exceeded 1 or if the score of a transverse sinus was 0, the stenoses were considered *lateralized* and assigned to the transverse sinus with the higher score.

### Primary outcomes

The primary objectives of the study were, first, to compare the four participant groups—healthy controls (Group A), patients with MwoA (Group B), patients with MwA (Group C) and IIH subjects (Group D)—with respect to each of the four radiological markers of raised ICP, namely the mean ECCS, the presence of an empty sella, optic-nerve diameter and posterior globe flattening. In addition, for the ECCS, the optimal cut-off distinguishing cases (Groups B-C-D) from healthy controls (Group A), dichotomizing the variable into ‘venous stenosis present’ versus ‘absent’, was calculated, allowing to subsequently compare the prevalence of venous stenosis across the same four groups.

### Secondary outcomes

Secondary outcomes included the comparison of the groups in terms of the frequency of bilateral venous stenosis, and the assessment of whether each radiological marker predicts monthly headache frequency.

## Statistical analysis

This is the primary analysis of these data. Descriptive analysis is reported for baseline variables, including frequency and percentage for categorical variables, and mean and standard deviation for continuous variables. Normality of distributions were assessed via the Shapiro-Wilk test. Continuous or ordinal variables that were not normally distributed have been reported as median and interquartile range.

The ECCS (range 0–10) was treated as ordinal. Global group differences were first explored with the Kruskal–Wallis H test. Whenever the global test reached significance (*α* = 0.05), pair-wise contrasts were obtained with Dunn's procedure and a Šidák adjustment for the six planned comparisons.

To translate the ordinal venous-impairment score into a binary diagnostic test, we performed an exploratory receiver operating characteristic analysis. The outcome variable was the ECCS; the state variable was a dichotomous flag identifying cases and controls. A non-parametric receiver operating characteristic curve was generated on the entire cohort, obtained the area under the curve with its 95% confidence interval. The optimal threshold was chosen by maximizing Youden's index (sensitivity + specificity − 1). To guard against over-fitting, an internal split-sample validation was carried out: 70% of the records were randomly assigned to a training set (selection based on a uniform random number without replacement) and the remaining 30% formed an independent test set. The Youden cut-off derived in the training data was applied unchanged to the test data.

After dichotomizing the ECCS score at the obtained threshold, the prevalence of the so obtained stenosis presence was compared across the four diagnostic groups. To locate pair-wise differences, Fisher's exact tests were performed between each subgroup, controlling for multiple comparisons with Šidák correction. The same statistical methodology was followed to compare the prevalence of bilateral stenosis between the four subgroups.

Likewise, other categorical outcomes—differences in the distribution of empty sella and flattening of the ocular bulb between subgroups—were tested with Fisher's exact tests, controlling for multiple comparisons with Šidák correction. Conversely, differences in optic nerve sheaths diameter were first assessed with a Kruskal–Wallis H test, with *post hoc* pairwise contrasts obtained with Dunn procedure, controlling for multiple comparisons with Šidák correction.

We exploratively assessed whether sex and headache burden acted as confounders for the four radiological outcome variables. Posterior globe flattening and empty sella, both binary, were modelled with standard logistic regression. The ordinal ECCS was analysed with proportional-odds logistic regression (logit link), while left and right optic-nerve diameters, continuous measures, were entered as the dependent variable in a multiple linear regression that additionally included the contralateral optic-nerve diameter as a covariate because of its strong anatomical correlation. Sex and the number of headache days per month were entered simultaneously in every model. Model fit was evaluated with the omnibus likelihood-ratio test and the Hosmer–Lemeshow statistic for binary outcomes, Pearson and deviance goodness-of-fit tests for the ordinal model, and inspection of residual plots and collinearity diagnostics for the linear model.

Finally, to test whether radiological signs of raised ICP predict headache burden, we fitted an explorative multiple linear regression model after excluding healthy controls so as to avoid the structural floor effect they introduce. The dependent variable was the monthly number of headache days. Five imaging predictors were entered simultaneously with the enter method: ECCS, empty sella sign, posterior globe flattening, and left and right eye optic-nerve diameters. Residual normality was assessed with normal-probability plots, homoscedasticity with plots of standardized residuals versus fitted values, and multicollinearity with tolerance and variance-inflation factors.

Given the paucity of existing data, no statistical power calculation was made prior to this study. No preregistered statistical analysis plan was available; analyses were primarily hypothesis driven.

Significance was set at *P* < 0.05, two sided. Data were managed and analysed in Matlab R2024a.

## Results

### Baseline and demographic data

A total 198 MRI/MRV examinations were eligible and included in the analysis: 37 from healthy controls, 89 from patients with MwoA, 36 from patients with MwA and 36 from patients with IIH. Main baseline characteristics are summarized in Table 2. There were no missing data. Cases comprised 120 females (75%) and 41 males (25%), whereas controls comprised 11 females (30%) and 26 males (70%). A Fisher's exact test confirmed a marked difference in sex distribution between groups (*P* < 0.001; OR = 6.92 (95% CI 2.14–15.23)). However, neither sex nor headache days showed an independent association with any radiological marker (for all, *P* > 0.05). Detailed statistic are reported in Supplementary Table 1.

**Table 2:** Baseline and demographic features

Variable	Controls (*n* = 37)	Migraine (*n* = 89)	Migraine aura (*n* = 36)	IIH (*n* = 36)
Age (years)	29 [25–32]	40 [31–51]	36 [25–44]	32.5 [19–43]
Sex (female)	11 (29.7%)	69 (77.5%)	27 (75.0%)	24 (66.6%)
Extended Combined Conduit Score	2 [1–3]	3 [2–4]	3 [3–4]	6 [5–6]
Episodic (*n*, %)	0 (0%)	34 (38.2%)	26 (72.2%)	16 (44.4%)
Chronic (*n*, %)	0 (0%)	55 (61.8%)	10 (27.8%)	20 (55.6%)
No pain (*n*, %)	37 (100%)	0 (0%)	0 (0%)	0 (0%)
Current preventive treatment	0 (0%)	89 (100%)	36 (100%)	36 (100%)
BMI (kg/m^2^)	—	—	—	27.1 (23.5–32.0)
Opening pressure (mmH_2_O)	—	—	—	286 [248–325]
Empty sella (*n*, %)	5 (13.5%)	35 (39.3%)	13 (36.1%)	29 (80.5%)
Flattening of the ocular bulb (*n*, %)	0 (0%)	11 (12.3%)	1 (2.7%)	18 (50.0%)
Optic nerve diameter, right (mm)	5.82 [5.44–6.01]	5.68 [5.20–6.39]	5.45 [5.15–5.97]	6.5 [6.2–7.2]
Optic nerve diameter, left (mm)	5.76 [5.42–6.13]	5.57 [5.06–6.20]	5.57 [5.13–5.97]	6.55 [6.00–7.3]

### Extended Combined Conduit Score

**Figure 1:**
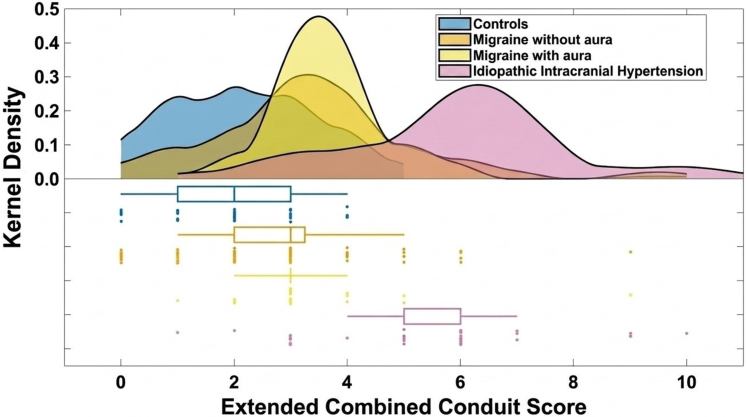
**Extended Combined Conduit Score across the four subgroups**. Each dot represents one participant. Median (IQR) ECCS: controls 2 (1–3, *n* = 37), MwoA 3 (2–4, *n* = 89), MwA 3 (3–4, *n* = 36), IIH 6 (5–6, *n* = 36). Kruskal–Wallis overall: H = 70.100, df = 3, *P* < 0.001. Dunn–Šidák *post hoc*: MwoA > controls Q = 3.437, *P* < 0.001; MwA > controls Q = 4.114, *P* < 0.001; IIH > controls Q = 8.147, *P* < 0.001; IIH > MwoA Q = 6.251, *P* < 0.001; IIH > MwA Q = 4.006, *P* < 0.001; MwoA versus MwA Q = 1.471, *P* = 0.141.

The ECCS showed a progressive increase across groups. Median and interquartile range values were 2 (1–3) in controls, 3 (2–4) in MwoA, 3 (3–4) in MwA and 6 (5–6) in IIH. The Kruskal–Wallis test confirmed a significant overall difference in the ECCS (H = 70.100, df = 3, *P* < 0.001). *Post hoc* Dunn–Šidák contrasts showed that median ECSS increased stepwise from controls < MwoA < MwA < IIH: all *post hoc P* < 0.001, with a single non-significant distinction between MwoA and MwA, *P* = 0.1413 (Fig. 1). Detailed pairwise statistics are reported in Supplementary Table 2.

### ECCS cut-off

On the full dataset of 198 examinations (161 patients, 37 controls), the ECCS discriminated cases from controls with an area under the curve of 0.786 (SE = 0.036, 95% CI 0.716–0.856, *P* < 0.001). The maximum Youden index (J = 0.475) occurred at a cut-off score of ≥3, that corresponded to sensitivity 0.745 and specificity 0.730.

### Sinus stenosis prevalence

**Figure 2:**
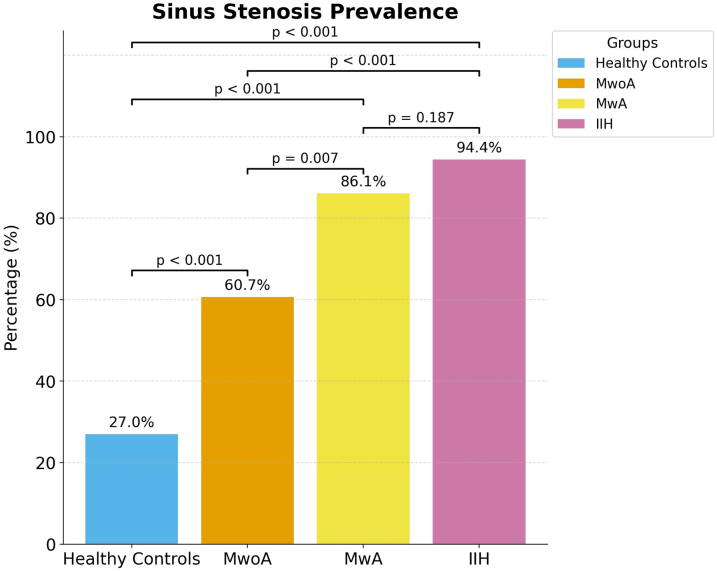
**Prevalence of an Extended Combined Conduit Score ≥3 across the four subgroups**. Prevalence: controls 27.0% (*n* = 37), MwoA 60.7% (*n* = 89), MwA 86.1% (*n* = 36) and IIH 94.4% (*n* = 36). Pairwise Fisher's exact tests (Šidák-adjusted): MwoA versus controls *P* < 0.001; MwA versus controls *P* < 0.001; IIH versus controls *P* < 0.001; IIH versus MwoA *P* < 0.001; MwA versus MwoA *P* = 0.007; MwA versus IIH *P* = 0.187.

After dichotomization at ≥3, sinus stenosis prevalence resulted 27.0, 60.7, 86.1 and 94.4% in Healthy Controls, MwoA, MwA and IIH groups, respectively (Fig. 2). Pairwise Fisher's exact tests produced *P* < 0.001 for MwoA versus controls, *P* < 0.001 for MwA versus controls, *P* < 0.001 for IIH versus controls, *P* < 0.001 for IIH versus MwoA, and *P* = 0.007 for MwA versus MwoA, while it did not reach significance (*P* = 0.187) for MwA versus IIH.

### Bilateral sinus stenosis prevalence comparison

**Figure 3:**
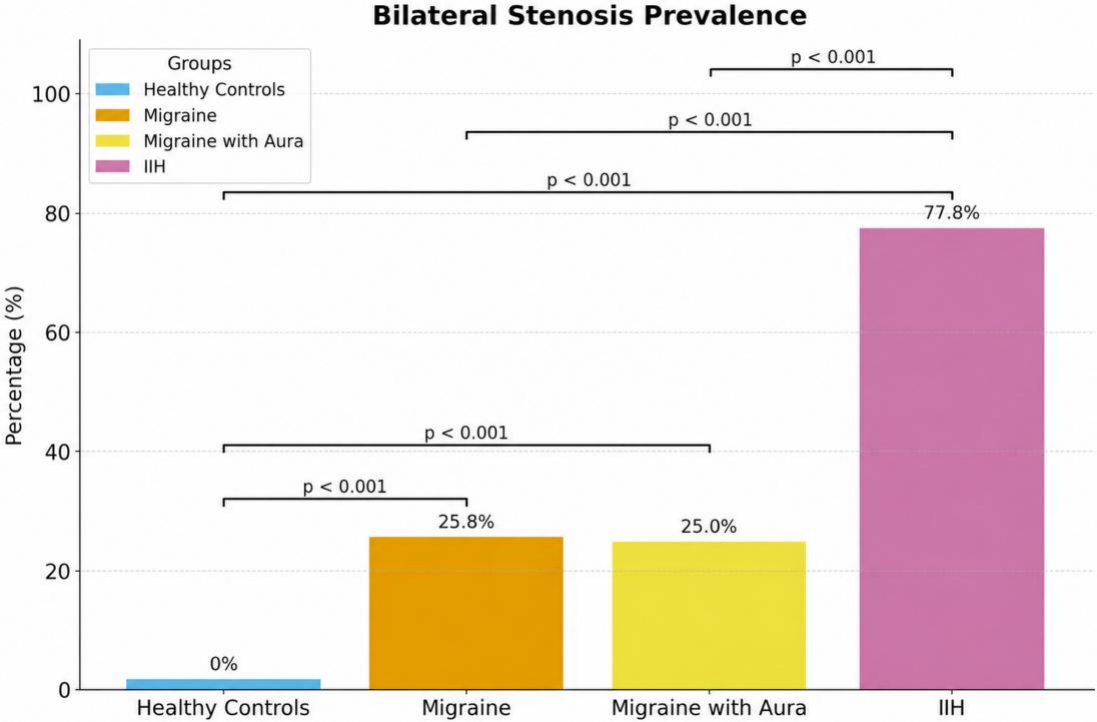
**Prevalence of bilateral sinus stenosis across the four subgroups**. Prevalence: controls 0% (*n* = 37), MwoA 25.8% (*n* = 89), MwA 25.0% (*n* = 36) and IIH 77.8% (*n* = 36). Pairwise Fisher's exact tests (two sided, Šidák-adjusted): MwoA versus controls *P* < 0.001; MwA versus controls *P* < 0.001; IIH versus MwoA *P* < 0.001; IIH versus MwA *P* < 0.001.

Bilateral transverse-sinus stenosis was present in none of the controls (0%) and in 25.8% of patients with MwoA (*P* < 0.001 versus controls) and 25.0% of those with MwA (*P* < 0.001 versus controls). The prevalence rose to 77.8% in the IIH group, a proportion significantly higher than in either migraine subgroup (*P* < 0.001) (Fig. 3).

### Empty sella

**Figure 4:**
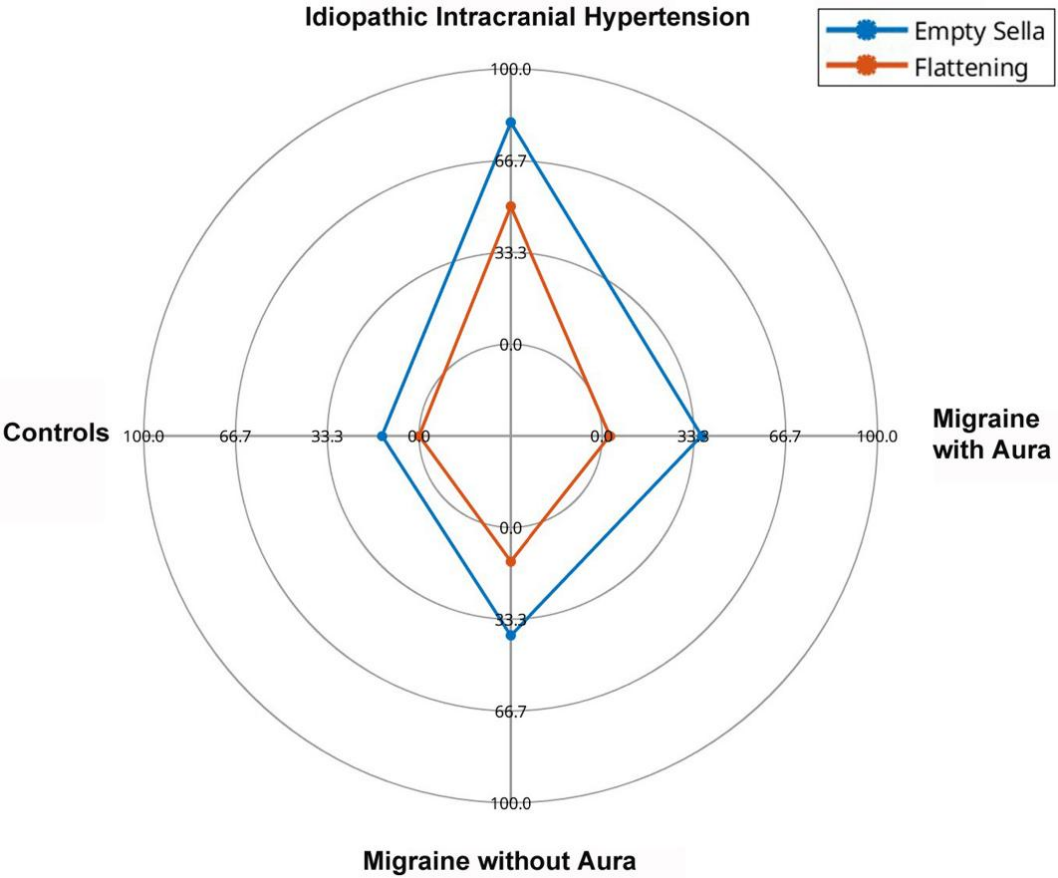
**Prevalence of empty sella and flattening of the posterior ocular globe across the four subgroups**. Empty sella prevalence: controls 13.5% (5/37), MwoA 39.3% (35/89), MwA 36.1% (13/36) and IIH 80.5% (29/36). Fisher's exact test with Lancaster correction: overall *P* < 0.001; Šidák-adjusted pairwise: controls versus IIH *P* < 0.001, controls versus MwoA *P* = 0.004, controls versus MwA *P* = 0.025; IIH versus MwoA *P* < 0.001, IIH versus MwA *P* < 0.001; MwoA versus MwA *P* = 0.764. Posterior globe flattening prevalence: controls 0% (0/37), MwoA 12.3% (11/89), MwA 2.7% (1/36) and IIH 50.0% (18/36). Fisher's exact test: overall *P* < 0.001; Šidák-adjusted pairwise: controls versus IIH *P* < 0.001, MwA versus IIH *P* < 0.001 and MwoA versus IIH *P* < 0.001; other comparisons not significant at Šidák α.

Empty sella was observed in 80.5% of IIH cases (29/36), 36.1% of MwA cases (13/36), 39.3% of MwoA cases (35/89) and 13.5% of controls (5/37). A Fisher's exact test with Lancaster correction confirmed highly significant differences across groups (*P* < 0.001). Šidák-adjusted pairwise comparisons showed that controls differed from IIH (*P* < 0.001), from MwoA (*P* = 0.004) and from MwA (*P* = 0.025), and that both migraine subgroups differed from IIH (each *P* < 0.001), whereas MwoA versus MwA did not differ (*P* = 0.7635) (Fig. 4).

### Flattening of the posterior globe

Flattening of the posterior globe was seen in 50.0% of IIH cases (18/36), 2.7% of MwA cases (1/36), 12.3% of MwoA cases (11/89) and 0% of controls (0/37). Fisher's exact test again showed a highly significant overall difference (*P* < 0.001). Šidák-corrected *post hoc* testing demonstrated that controls versus IIH cases (*P* < 0.001), MwA versus IIH cases (*P* < 0.001) and MwoA versus IIH cases (*P* < 0.001) were all significant, while controls versus MwoA; MwoA versus MwA; and controls versus MwA did not because their *P*-values were greater than Šidák alphas (Fig. 4).

### Optic nerve sheats diameters

**Figure 5:**
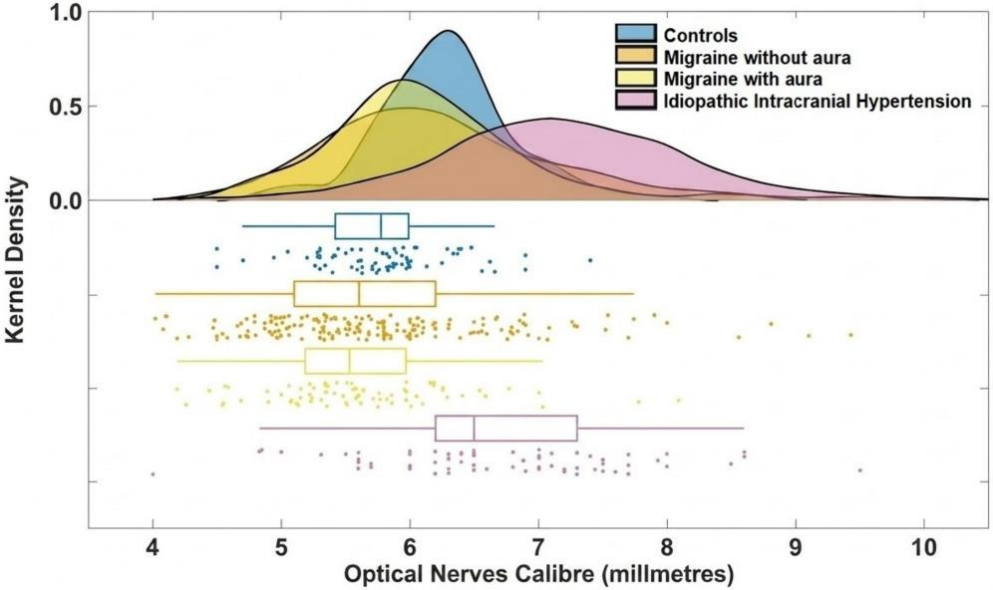
**Optic nerve sheaths diameters across the four subgroups.** Each dot represents one optic nerve measurement; right and left optic nerves are displayed as separate observations. Participant numbers were: controls *n* = 37, MwoA *n* = 89, MwA *n* = 36 and IIH *n* = 36. No lateral asymmetry within groups (Monte Carlo/randomization and exact tests *P* = 0.224–1.000). Between-group comparison (pooled per participant): Kruskal–Wallis χ^2^ = 65.311, df = 3, *P* < 0.001. Dunn–Šidák *post hoc*: IIH > controls *P* < 0.001, IIH > MwoA *P* < 0.001, IIH > MwA *P* < 0.001; MwoA versus MwA not significant (Šidák α).

Optic nerve sheaths diameter showed no evidence of lateral asymmetry in any group: Monte Carlo randomization and exact tests yielded *P*-values ranging from 0.224 to 1.000, confirming bilateral symmetry (*P*-value range: 0.224–1.000). When comparing median diameters between groups, the Kruskal–Wallis test demonstrated a highly significant overall difference (χ^2^ = 65.311, df = 3, *P* < 0.001). *Post hoc* Dunn–Šidák tests revealed that only the IIH group had significantly larger diameters versus controls (*P* < 0.001), MwoA (*P* < 0.001) and MwA (*P* < 0.001); no significant difference was observed between the two migraine subgroups (*P* > Šidák alphas) (Fig. 5).

### Impact of radiological signs on monthly headache days

The model included 161 patients (IIH and migraine cohorts only). Overall fit was poor (*R* = 0.141; *R*^2^ = 0.020; adjusted *R*^2^ = −0.012), and the predictors jointly failed to account for a significant proportion of the variance in headache days (*P* = 0.679). None of the individual imaging variables reached statistical significance: ECSS (B = −0.23 ± 0.51 days per point, *P* = 0.648), empty sella (B = −1.50 ± 1.89 days, *P* = 0.430), globe flattening (B = −2.68 ± 2.62 days, *P* = 0.308), left optic-nerve diameter (B = 0.86 ± 1.91 days · mm^−1^, *P* = 0.653) and right optic-nerve diameter (B = −0.51 ± 1.77 days · mm^−1^, *P* = 0.775).

## Discussion

### Extended Combined Conduit Score validation

The receiver operating characteristic curve analysis of the proposed ECCS for grading dural sinus stenosis, modified by Farb^48^ to include the superior sagittal sinus and the separation at the torcular Herophili, supports the value of ≥3 as a possible cut-off between normal and pathological conditions. This implies that even moderate and unilateral narrowing of dural sinuses—currently regarded almost universally as normal anatomical variants—may already impair ICP regulation in a clinically significant way.

### Migraine without aura, migraine with aura, and IIH: a pathogenetic continuum

The main findings of our study indicate that in both migraine groups—without and with aura (Groups B and C)—the prevalence and grading of venous stenosis and the frequency of empty sella, representing the two non-ophthalmological neuroradiological signs of IIH, were significantly higher than in healthy controls, though still lower than in the IIH group (Group D). Furthermore, in Group C (MwA), the prevalence of cases with a ECCS ≥3 was significantly higher than in Group B (MwoA), and not significantly different from Group D with IIH. The median ECSS did not distinguish between migraine with and without aura but did separate both from healthy controls and IIH patients. Notably, patients with IIH had a prevalence of stenosis ≥3 comparable to patients with MwA, but was characterized by a much higher frequency of bilateral stenoses, higher mean stenosis scores, and, as expected, a greater prevalence of ophthalmologic neuroradiological signs: optic nerve sheath enlargement and globe flattening.

Taken together, these data suggest that a derangement of ICP, likely of moderate degree or intermittent course—and therefore insufficient to generate significant optic nerve sheath enlargement, globe flattening, and especially papilledema—may distinguish migraine patients, particularly those with aura, from healthy controls on one side and from those with defined IIH on the other. This is best conceptualized within a pathophysiological framework of fluid-dynamic dysregulation, without necessarily corresponding to current diagnostic criteria of IIH or IIHWOP.^6^

This raises the hypothesis of a pathogenetic *continuum* between migraine and IIH, potentially explaining their widely documented similarities in clinical presentation,^7,8^ shared risk factors,^10,11^ allodynic symptoms,^12,13^ sinus stenosis prevalence,^15-18^ pathogenetic involvement of CGRP,^27,28^ and response to various treatments, including topiramate,^14^ anti-CGRP/R monoclonal antibodies,^26^ GLP-1 receptor agonists^29,30^ and lumbar puncture with CSF withdrawal.^20,21^

### Papilledema: from diagnostic cornerstone to infrequent complication

It is well established that bilateral venous sinus stenosis is a strong predictor of classical IIH with papilledema.^51^ Our data confirm this notion, showing that bilateral stenoses, already significantly more prevalent in migraine patients without (25.8%) and with aura (25.0%) than in controls (0%), reached a prevalence of 77.8% in the IIH group, much higher than in both migraine groups. Therefore, considering the striking prevalence gap between the very common migraine and the rare IIH, our findings raise the question of whether papilledema reflects severity rather than presence of ICP elevation, possibly occurring in the most severe cases of cerebral venous outflow obstruction characterized by bilateral venous stenosis—this will naturally require prospective validation. Among the countless consequences of this assumption, there is the potential to downgrade obesity from a possible dysmetabolic causative factor of IIH^52^ to a powerful risk factor for papilledema development. Increased body weight significantly raises central venous pressure, and thus dural sinus pressure,^5,53^ creating the conditions for development of papilledema in the minority of obese individuals with pre-existing sinus stenoses. This perspective could justify the strong association between IIH and obesity on fluid dynamic rather than metabolic grounds and explain why, in a recent longitudinal prospective cohort study, no significant difference could be found—either at baseline or along median 17 months observation period—in visual acuity, papilledema or macular ganglion cell layer volume, between overweight or obese and normal weight IIH patients.^54^

### What is the true prevalence of IIHWOP?

Our findings suggest that the current clinical and pathogenetic understanding of IIH—largely derived from cohorts selected based on the presence of papilledema—may represent only the most severe end of the disease spectrum, rather than its true prevalence and full range of clinical phenotypes. This conclusion is supported by several considerations: the high prevalence of significant venous sinus stenoses associated with elevated ICP in individuals lacking ‘persistent’ headache or other typical IIH signs or symptoms—up to 11.1%, according to Bono and colleagues;^55^ a reduced intracranial compliance even unassociated with pathological opening pressure values may be pathological itself as it may cause organ damage^53^ or invalidating chronic fatigue syndrome, responding to CSF subtraction by lumbar puncture^56^ or to sinus stenting;^57^ the pathogenic role of these stenoses, evidenced not only by the clinical efficacy of stenting procedures,^58^ but also by data showing that even a one-third reduction in dural sinus calibre can generate a pathological venous pressure gradient;^59^ and the tendency of most neuroradiologists to dismiss even prominent stenoses as ‘normal variants’. This common diagnostic bias probably stems and is perpetuated by a ‘circular reasoning’ between clinicians and radiologists, as insightfully pointed out by Higgins.^60^ Taken together, these factors generate the hypothesis that disturbances in CSF volume and pressure regulation—although moderate or intermittent and thus insufficient to cause papilledema or meet the low-sensitivity criteria for IIHWOP proposed by Friedman and colleagues^6^—which still require an opening pressure >250 mm H_2_O and the presence of sixth nerve palsy if papilledema is lacking—may in fact be several orders of magnitude more prevalent than currently recognized.

### Intracranial hypertension without papilledema and migraine: risk factor or co-causative factor?

The relationship between elevated ICP and headache is complex. On the one hand, reduction of ICP through sinus stenting, CSF diversion or even a single lumbar puncture with CSF withdrawal is usually followed by immediate improvement or resolution of headache, confirming a causal link between elevated ICP and headache.^20,21,58,61^ On the other hand, the IIHTT study^9^ showed that 15.7% of IIH patients do not report headache; in about 25% of cases the headache resembles a tension-type or unclassified headache; and a migraine-like phenotype appears in about 67% of cases. Furthermore, only about half of these headaches were chronic (>15 days/month), with the rest experiencing a mean headache frequency of 12 days/month. Notably, the study also found no difference in mean opening pressure between those with and without headache and no correlation between opening pressure values and attack frequency. Furthermore, there is evidence that headache may persist despite papilledema resolution.^62^ Rather than indicating a lack of causal effect these findings may suggest, on one hand, that the ICP threshold for headache triggering is far lower than that that required for papilledema development and, on the other, that raised ICP could be a necessary but not sufficient condition for pain generation. Likely, an individual predisposition to headache, especially migraine, is also necessary and may influence not only the presence or absence of pain but also the clinical phenotype and frequency of attacks.

Our data support this hypothesis, as no predictors of increased headache frequency were found in the regression model in regards to venous impairment, flattening of the posterior ocular globe, empty sella, or optic nerve sheath enlargement in our patients. This suggests that ICP-related imaging signs may point to a background predisposition to develop migraine, rather than directly influence attack frequency. In other words, headache development in the presence of altered ICP may always be modulated by an individual susceptibility to head pain, likely dependent by other factors, including genetic ones. Supporting this interpretation is the well-established observation that experimental administration of CGRP or pituitary adenylate cyclase-activating peptide triggers attacks only in migraineurs.^63,64^

### Is migraine a primary or a secondary disease?

The above considerations and data suggest that impaired intracranial volume and pressure regulation due to disrupted cerebral venous outflow could represent a necessary, though not sufficient, condition for migraine development, and an important novel therapeutic target for this widespread and highly disabling disorder.^30^ Following this perspective, migraine with and without aura remains a condition defined by a ‘primary’ predisposition to migraine pain. The nature of this predisposition remains unknown, but it is likely multifactorial and varies widely among individuals and in the same individual over time. One key factor could be cyclical hormonal fluctuations in females. A reduction in trigeminal allodynic threshold has been documented in women during the menstrual phase after subcutaneous capsaicin injection.^65^ IIH has been reported to run a headache-free course in a patient without any migraine family history and in one case during pregnancy, a condition known to be protective against migraine.^66^

In this context, migraine remains a ‘primary’ condition, but confined to a subgroup of individuals sharing impaired ICP regulation due to disturbed cerebral venous outflow.

### Study limitations

Healthy controls had a male predominance (70%) and a significantly lower percentage of females (30%). Although statistical analysis ruled out an influence of this imbalance on the study outcomes, it remains possible that the control group does not reflect a representative sample of the general population, potentially affecting data interpretation, particularly for conditions like migraine and IIH where sex is a key determinant. However, the male preponderance might have led to the inclusion of asymptomatic cases in the control group, without affecting the strength of the data. The control group was drawn from an ongoing parallel study on dural sinus anatomy in healthy individuals, where volunteer recruitment has been challenging.

We did not directly measure ICP measurements in migraine cohorts, limiting definitive inferences about pathophysiology, and BMI data were available only for IIH, precluding adjustment for this confounder across all models. The ECCS ≥3 threshold was derived and evaluated within the same dataset, so diagnostic performance should be viewed as exploratory. In addition, the single-centre setting recruitment limits generalizability. Some covariates were not systematically captured (e.g. hormonal status, medication use, sleep-related factors), which could influence both venous outflow and headache phenotypes. The IIH cohort was small, which reduces power to detect differences versus migraine subgroups and limits generalizability, and the tertiary-referral sample may overrepresent more severe IIH, introducing referral bias that could affect the magnitude and direction of group contrasts. Taken together, these caveats warrant cautious interpretation and motivate prospective, multicentre validation, while the present data provide a coherent signal that can guide hypothesis refinement and future study design.

The lack of correlation between stenosis severity and the presence or frequency of painful episodes supports the idea that ICP dysregulation is necessary but not sufficient for migraine development. However, the low-frequency migraine cases included here were clinically, not population-based, and may represent more severe or treatment-resistant cases referred to a tertiary centre. We cannot exclude that lower-impact episodic migraine, responsive to over-the-counter treatments and thus not represented in our sample, may have a lower prevalence of venous sinus stenoses and other neuroradiological signs of elevated ICP, potentially similar to healthy controls.

Another limitation concerns the measurement of optic nerve diameters and globe flattening. In some cases, the orbital MRI slice available did not coincide with the maximum optic nerve diameter, leading to underestimation and affecting visualization of flattening. Future studies should include optic nerve diameter measurement using A-mode ultrasound, which allows hundredths-of-a-millimetre precision.

## Conclusions

Three major and mutually reinforcing biases in the medical literature—papilledema as the hallmark of IIH; the alleged rarity of IIHWOP; and the presumed pathophysiological irrelevance of milder or unilateral dural sinus stenoses—have hindered recognition of the critical pathogenetic role of altered cerebral venous outflow in the dysregulation of CSF volume and pressure control. Our findings indicate that papilledema, if anything, could represent the endpoint rather than the starting point of the disease, characterizing only a minority of severe IIH cases; a form of IIHWOP not meeting current diagnostic criteria could be much more prevalent than believed and potentially implicated in both chronic and episodic migraine; and dural sinus calibre alterations, even if moderate or unilateral, should not be dismissed as ‘normal variants’ but seen as key contributors to varying degrees of intracranial volume and pressure control dysfunction.

This implies the need to revise the very definition of IIH and its diagnostic criteria, which are currently calibrated only to the possibly small subset of patients developing papilledema, thereby likely perpetuating high rates of misdiagnosis or non-recognition of papilledema-free forms and their true phenotypes and clinical trajectories.

Overall, our findings generate the hypothesis that an altered ICP regulation associated with predominantly unilateral venous sinus stenosis could be involved with both chronic and episodic migraine and may explain the remarkable clinical, pathogenetic, and therapeutic overlap between migraine and migraine-like IIH headache. Further research should aim to assess whether ICP control, possibly by preventing CGRP release, could be considered as a novel therapeutic target for migraine, either as an alternative or as a complement to CGRP antagonists or CGRP receptor blockers.

## Supplementary Material

fcag259_Supplementary_Data

## Data Availability

The data that support the findings of this study are available from the corresponding author, upon reasonable request. The codes generated for the analyses in this manuscript are available at https://github.com/dnafinder.
