# Supplementary material for: Papilledema is not the point: intracranial pressure dysregulation as a common pathway in migraine and idiopathic intracranial hypertension
Source: Brain Commun. 2026 Jul 7;8(4):fcag259. doi: 10.1093/braincomms/fcag259 (PMC13457942; doi:10.1093/braincomms/fcag259)
Supplement: Supplementary file 1 [file fcag259_Supplementary_Data.docx]

**Supplementary Material**

Supplementary Table 1 — Regression models (sex & baseline headache days) on radiological parameters

| **Outcome** | **Model** | **Predictor** | **Effect size** | **95% CI** | **p-value** |
| --- | --- | --- | --- | --- | --- |
| Posterior globe flattening | Logistic | Sex (M vs F) | OR 0.89 | 0.38–2.11 | 0.794 |
| Posterior globe flattening | Logistic | Baseline headache days (per day) | OR 0.996 | 0.96–1.03 | 0.827 |
| Empty sella | Logistic | Sex (M vs F) | OR 1.15 | 0.61–2.17 | 0.675 |
| Empty sella | Logistic | Baseline headache days (per day) | OR 1.02 | 0.99–1.04 | 0.289 |
| ECCS (ordinal) | Proportional-odds | Sex (M vs F) | OR 1.55 | 0.89–2.71 | 0.120 |
| ECCS (ordinal) | Proportional-odds | Baseline headache days (per day) | OR 1.01 | 0.99–1.04 | 0.246 |
| Optic nerve diameter — Left | Linear | Sex (M vs F) | B = 0.02 mm | −0.12 to 0.17 | 0.772 |
| Optic nerve diameter — Left | Linear | Baseline headache days (per day) | B = −0.00008 mm | −0.006 to 0.006 | 0.979 |
| Optic nerve diameter — Right | Linear | Sex (M vs F) | B = 0.077 mm | −0.069 to 0.224 | 0.299 |
| Optic nerve diameter — Right | Linear | Baseline headache days (per day) | B = 0.001 mm | −0.005 to 0.007 | 0.734 |

Supplementary Table 2 — Pairwise Dunn–Šidák contrasts for ECCS

| **Comparison** | **Q statistic** | **Adjusted p-value** | **Significance** |
| --- | --- | --- | --- |
| MwoA vs Controls | 3.437 | <0.001 | Yes |
| MwA vs Controls | 4.114 | <0.001 | Yes |
| IIH vs Controls | 8.147 | <0.001 | Yes |
| IIH vs MwoA | 6.251 | <0.001 | Yes |
| IIH vs MwA | 4.006 | <0.001 | Yes |
| MwoA vs MwA | 1.471 | 0.1413 | No |
